# On mechanical behaviors of few-layer black phosphorus

**DOI:** 10.1038/s41598-018-21633-1

**Published:** 2018-02-19

**Authors:** Lili Li, Jie Yang

**Affiliations:** School of Engineering, RMIT University, PO Box 71, Bundoora, VIC 3083 Australia

## Abstract

This paper investigates the mechanical behaviors of few-layer black phosphorus (FLBP) by using molecular dynamics simulations. Results show that both tensile and compressive behaviors are strongly anisotropic in the armchair and zigzag directions due to the unidirectional puckers in each atomic layer, and that the compressive behavior is dependent on the number of atomic layers. In particular, the compressive and buckling strengths of FLBP can be significantly enhanced by stacking more atomic layers together, while this has little influence on both Young’s modulus and tensile strength. It is interesting to found that increasing the number of atomic layers in FLBP or the dimension ratio can lead to a drastically reduced flexibility in armchair direction, showing that both compressive and buckling strengths become higher than those in zigzag direction. It is also demonstrated that the reorientation of FLBP’s atomic configuration occurs under certain conditions. The mechanism of deformation underlying the mechanical behaviors of FLBP is also discussed, suggesting that changing the number of atomic layers is an effective way to engineer two-dimensional materials for desired material properties.

## Introduction

Recently, black phosphorus (BP) has been rediscovered from the perspective of a two-dimensional (2D) material and rapidly attracted tremendous interests due to its unique and superior electrical, optical and thermal properties^1–12^. Different with other 2D layered materials, BP is stacked by atomic P layers with unidirectional puckered microstructure, giving rise to strongly anisotropic properties. In addition, BP possesses intrinsic direct bandgap^3–5^ which is unavailable in graphene. More interestingly, such bandgap can be modified in a range of ~0.3 eV-2.0 eV by changing the number of atomic layers^6,7^ and/or sustained strains of the structure^8,9^. Due to these peculiar properties, the potential of integrating BP for technological application in nanoelectronic and nanophotonic fields has been readily envisioned^3–12^. For example, recent studies^3–5^ reported that BP is an appealing candidate for tuneable photodetection accessing a wide spectrum ranging from visible to infrared regime. Moreover, previous reports also indicated that BP is an outstanding semiconductor material^4–11^ and a promising alternative electronic material to graphene, MoS_2_, BN and so on for transistor applications^5–8^.

When tuning the electrical and optical properties of BP by applying external strain, it is necessary to understand its mechanical behaviors. So far, the majority of the existing studies on BP are primarily focused on its electrical and optical properties. Research work on mechanical attributes of few-layer black phosphorus (FLBP) is still rather limited although a few works on single-layer black phosphorus (SLBP) have been reported by theoretical^7,13–19^ and experimental studies^4^. By using first principle calculations, Jiang *et al*.^13^ discovered apparent negative Poisson’s ratio in SLBP when subjected to uniaxial stretching in the pucker (zigzag) direction, while positive Poisson’s ratio was observed when a uniaxial stress was applied in the perpendicular (armchair) direction. Moreover, Young’s modulus can vary by a factor of ~4 in the two orthogonal in-plane directions (*E*_zigzag_ ≈ 58.6–159 GPa and *E*_armchair_ ≈ 19.5–41.3 GPa)^4,7,14–17^. Our previous work^18^ demonstrated the role of prestrain on deformation behaviors in both two in-plane directions of SLBP at low temperature, showing that the armchair-oriented prestrain improves the modulus more significantly than the zigzag-oriented prestrain does. Wang *et al*.^19^ reported the anisotropic flexibility in the out-of-plane direction of SLBP and observed that curvatures emerged during compression, remaining the structure integrity as further compression is applied along the armchair direction whereas the structure breaks at a large strain in the zigzag direction.

The mechanical behavior of FLBP, however, has not been explored yet. Motivated by this, the present paper investigates how the tensile and compressive behaviors of FLBP are influenced by the total number of atomic layers *N*_*L*_ *via* MD simulations. The studies show that the compressive behavior of FLBP is strongly dependent with both crystallographic orientation and total number of atomic layers. Particularly, the reorientation emerges in FLBP before yielding during compression in the armchair direction while tensile behavior is not influenced by the number of atomic layers.

### Model and computational method

**Figure 1:**
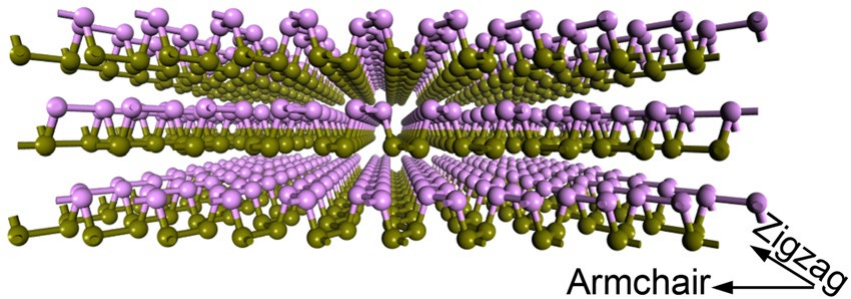
Atomic structure of a three-layer BP crystal.

Figure 1 shows the atomic structure of FLBP with three atomic layers (*N*_*L*_ = 3), where the unidirectional puckers differentiate the two orthogonal in-plane directions, namely zigzag and armchair directions that are parallel and perpendicular to the puckers, respectively. The dimension of the unit cell is 103 Å × 103 Å, while the thickness varies in different cases. Periodic boundary conditions are applied in both armchair (*x*) and zigzag (*y*) directions while the free surface boundary is used in the out-of-plane (*z*) direction.

The MD algorithm used here is implanted in the LAMMPS code^20^ to simulate the uniaxial deformation of FLBP by applying tensile/compressive strain in the zigzag and armchair directions. The initial FLBP structure first undergoes energy minimization with the conjugate gradient method before running MD. The system was then equilibrated at NPT ensemble for 50 ps for internal stresses to be fully released. After that, an uniaxial tensile/compressive strain with a constant strain rate of 10^−4^ ps^−1^ is applied in either armchair (${\varepsilon }_{x0}$) or zigzag (${\varepsilon }_{y0}$) direction by increasing/decreasing the periodic simulation box size step by step in the corresponding direction while all atomic positions are remapped following the migration of the boundaries. During the MD simulation, the time step 1 fs is used to integrate the atomic motion equations. The bonds among P atoms in FLBP are calculated by using Stillinger-Weber potential which was recently developed for BP^15,16^. This potential is parameterized based on the valence force field and the computed phonon spectrum. The mechanical properties of BP thus obtained agree quite well with *ab initio* calculations^16^. The interaction between layers is described by Lennard-Jones potential parameterized for FLBP^21^. The thickness of a single layer is taken as 5.24 Å^16^ in the computation of atomic stress in the system. The thermal environment condition is considered to be a range from 1 K to 400 K simulated by a Nosé-Hoover^22,23^ thermostat.

## Results and Discussions

### Compressive behaviour

**Figure 2:**
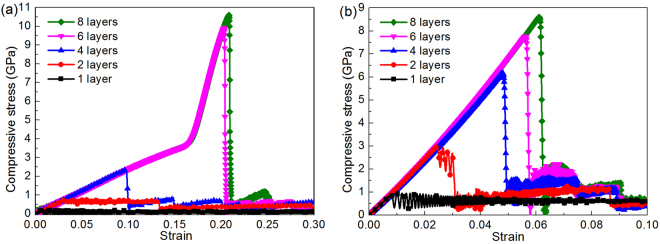
Compressive stress-strain curves of FLBP with multiple atomic layers in the (**a**) armchair and (**b**) zigzag directions.

The compressive behavior of FLBP with varying number of atomic layers is first examined. Figure 2 shows the compressive stress-strain response curves in the armchair and zigzag directions with the number of atomic layers *N*_*L*_ = 1, 2, 4, 6, 8, respectively. For better illustration, the curves are limited to compressive strain ε^−^ ≤ 0.3 in the armchair direction and ε^−^ ≤ 0.1 in the zigzag direction. As can be observed, the compressive behavior is strongly dependent on both *N*_*L*_ and crystallographic orientation. For SLBP, the compressive stress increases linearly all the way up to the critical point (i.e. compressive strength), followed by a near-flat regime where plastic deformation occurs. As compression continues, such regime sustains and no abrupt drop occurs in the stress-strain curve till the compressive strain reaches the ultimate strain (i.e. ~0.860 and ~0.491 in the armchair and zigzag directions). As *N*_*L*_ increases, the plateau becomes much shorter when *N*_*L*_ = 2 and even disappears when *N*_*L*_ > 2. Figure 2 also shows that for FLBP with four and more atomic layers (*N*_*L*_ ≥ 4), the stress drops abruptly beyond the ultimate strain. Among the FLBPs considered, SLBP has the highest ultimate strain while that of the FLBP with *N*_*L*_ ≥ 4 is much lower. This is due to the fact that SLBP is more flexible in the out-of-plane direction and can form a folded structure due to compression, which enables SLBP to sustain a large compressive strain^18,19^ while with more atomic layers stacked together, the out-of-plane flexibility is significantly decreased due to the interaction between atomic layers. For example, FLBP with *N*_*L*_ = 4 fails at ε^−^ = ~0.100 and ε^−^ = ~0.050 only in the armchair and zigzag direction, respectively. It is interesting to observe from Fig. 2a that for an FLBP with *N*_*L*_ = 6 and *N*_*L*_ = 8 compressed in the armchair direction, the slope of the curves which are both almost linear shows a sudden and significant increase after ε^−^ = ~0.165, indicating the “hardening” phenomenon after this point caused by higher interfacial interactions. This is different from the observations in Fig. 2b for FLBPs under compression in the zigzag direction, which show a linear stress-strain relationship up to the point where FLBP fails abruptly.

**Figure 3:**
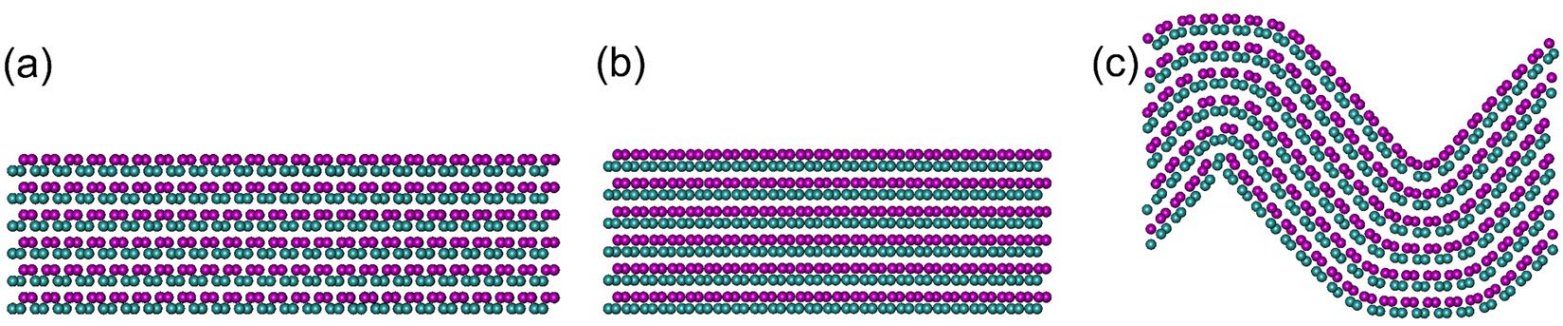
Snapshot of six-layer BP at selected compressive strains: (**a**) 0.000, (**b**) 0.165 and (**c**) 0.205 during compression process in the armchair direction.

**Figure 4:**
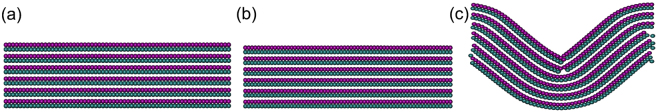
Snapshot of six-layer BP when compressing in the zigzag direction at selected compressive strains: (**a**) 0.000, (**b**) 0.020 and (**c**) 0.058.

To better understand the above-mentioned distinct stress-strain behavior, the deformed atomic configurations of FLBP in the armchair and zigzag directions at selected compressive strains are captured in Figs 3 and 4, respectively. At the initial state shown in Fig. 3a, the distance between two P atoms is much larger than that in the zigzag direction^15,16^. This distance becomes quite small and is just comparable with the distance in the zigzag direction when the compression strain is 0.165 as shown in Fig. 3b, making the compressive stress-strain response in the armchair direction become similar to that in the zigzag direction with a considerably bigger slope in the stress-strain curve. As compression continues, FLBP fails at a strain of ε^−^ = ~0.205, corresponding to the sudden drop in the stress-strain curve in Fig. 2a. When the FLBP is compressed in the zigzag direction as shown in Fig. 4, the zigzag chain continues folding till abrupt failure happens at ε^−^ = ~0.058. This corresponds to the compressive stress-strain response in Fig. 2b where stress is seen to linearly increase with strain and the curve suddenly drops beyond the ultimate stress.

It is worthy of noting that FLBP becomes slightly thicker under the compression in the armchair direction but tends to be thinner when compressed in the zigzag direction, as can be seen from direct comparisons between Figs 3b and 4b. This distinct behavior is attributed to the anisotropic Poisson’s ratio which is positive in the armchair direction and negative in the zigzag direction^13^ hence the compression in the armchair (zigzag) direction makes FLBP expand (contract) in the out-of-plane direction.

**Figure 5:**
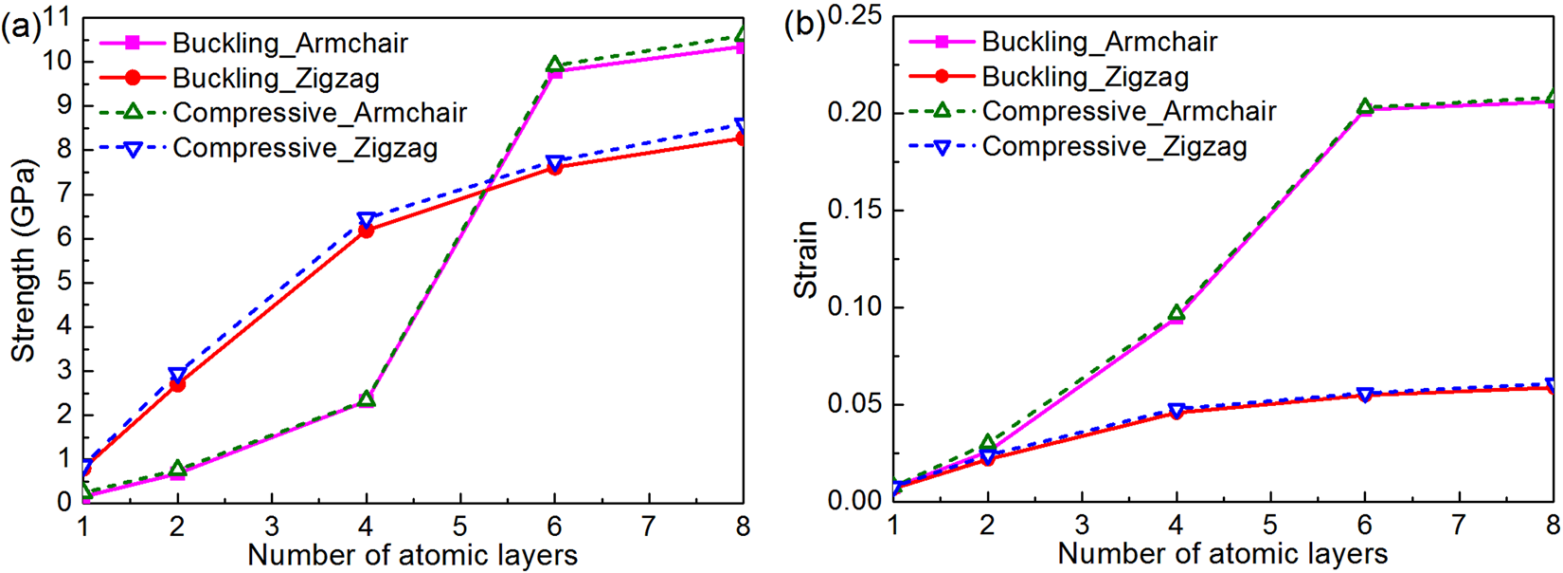
Effect of total number of atomic layers on FLBP’s compressive behaviors in both armchair and zigzag directions: (**a**) Buckling and compressive strengths; (**b**) Buckling and compressive strains.

The compressive modulus can be extracted from the slope of the compressive stress-strain curves in the initial small strain region (ε^−^ ≤ 0.01) using linear regression. Results in Table 1 shows that FLBP’s compressive moduli in both armchair and zigzag directions are not sensitive to the total number of layer with its value between 23.25~24.81 GPa in the armchair direction and 111.72~117.22 GPa in the zigzag direction, respectively. Moreover, by tracking the atomic motion trajectory during compression process, the buckling stress (strain), defined as the critical stress (strain) at which BP buckles, can be determined from stress-strain response. Figure 5 further depicts the effect of the total number of atomic layers on the compressive and buckling strengths and strains in both armchair and zigzag directions. As can be seen, compressive strength can be considerably improved by increasing the total number of atomic layers owing to the interaction between atomic layers which makes FLBP stiffer with a lower out-of-plane flexibility, thus leading to enhanced compressive and buckling strengths and strains. It is also interesting to note that the compressive and buckling strengths in the armchair direction overtake their counterparts in the zigzag direction when there are six and more atomic layers stacked together in the FLBP. Moreover, the buckling strength is just slightly lower than the compressive strength, indicating that the FLBP will fail very soon after buckling deformation happens.

**Table 1:** Compressive moduli (GPa) of FLBP with different atomic layers in two in-plane directions.

	1 layer	2 layers	4 layers	6 layers	8 layers
Armchair	24.81	23.25	24.14	24.06	23.60
Zigzag	111.72	116.09	116.84	116.98	117.22

Results in both Table 1 and Fig. 5 also clearly demonstrate distinct material properties in FLBP’s two in-plane directions. This is due to the anisotropic atomic structure (shown in Fig. 1) in which P atoms are arranged in a puckered lattice in the armchair direction which offers excellent capability of sustaining a higher external strain through change in pucker angle instead of bond length^18,19^, resulting in a lower compressive and buckling strength but a larger compressive strain. On the contrary, the zigzag chain-like lattice for P atoms in the zigzag direction limits the flexibility and makes FLBP fail at a relatively small strain but with a higher strength in this direction.

**Figure 6:**
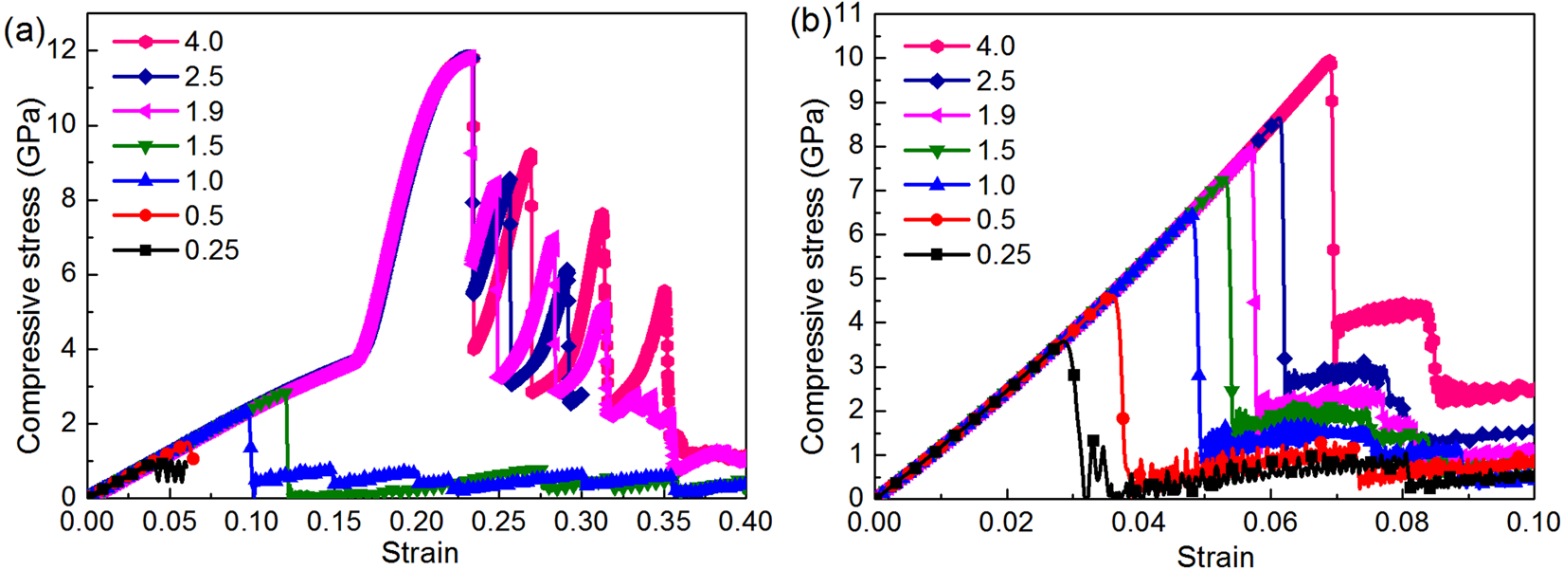
Effect of *L*_a_/*L*_b_ ratio on FLBP’s stress-strain curves when compressed in (**a**) armchair and (**b**) zigzag directions. The numerical numbers in legend denote *L*_a_/*L*_b_ ratio.

Considering the fact that FLBP-based electronic devices are fabricated in various geometric dimensions, it is essential to understand how the compressive and buckling strengths of FLBP are affected by its dimensions. By taking a four-layer BP as an example and keeping its thickness and volume (total number of P atoms in the model) constant while varying its in-plane dimensions, the dimension-dependent compressive behaviors in both armchair and zigzag directions are investigated in Fig. 6 which shows the compressive stress-strain response in the two directions with different *L*_a_/*L*_b_ ratios. Here *L*_a_ and *L*_b_ denote the length of the side perpendicular and parallel to the loading direction, respectively. As a special case, *L*_a_/*L*_b_ = 1 corresponds to a square shaped FLBP. It can be seen that the compressive strength increases as *L*_a_/*L*_b_ ratio increases. When the compression is applied in the zigzag direction, the stress-strain behaviors in Figs 2b and 6b are quite similar but for a rectangular FLBP with a high *L*_a_/*L*_b_ ratio (e.g. *L*_a_/*L*_b_ ≥ 1.9) compressed in the armchair direction, the slope (i.e. compressive Young’s modulus) of the stress-strain curve exhibits a sudden and remarkable jump at ε^−^ = ~0.165 and becomes that of the stress-strain curve under a compression in the zigzag direction, as shown in Fig. 5a. Such phenomenon has also been observed in the compressive test in Fig. 2a for FLBP with a high number of atomic layers (i.e. *N*_*L*_ ≥ 4), suggesting that the armchair lattice of FLBP can shift to the zigzag configuration under a certain condition.

**Figure 7:**
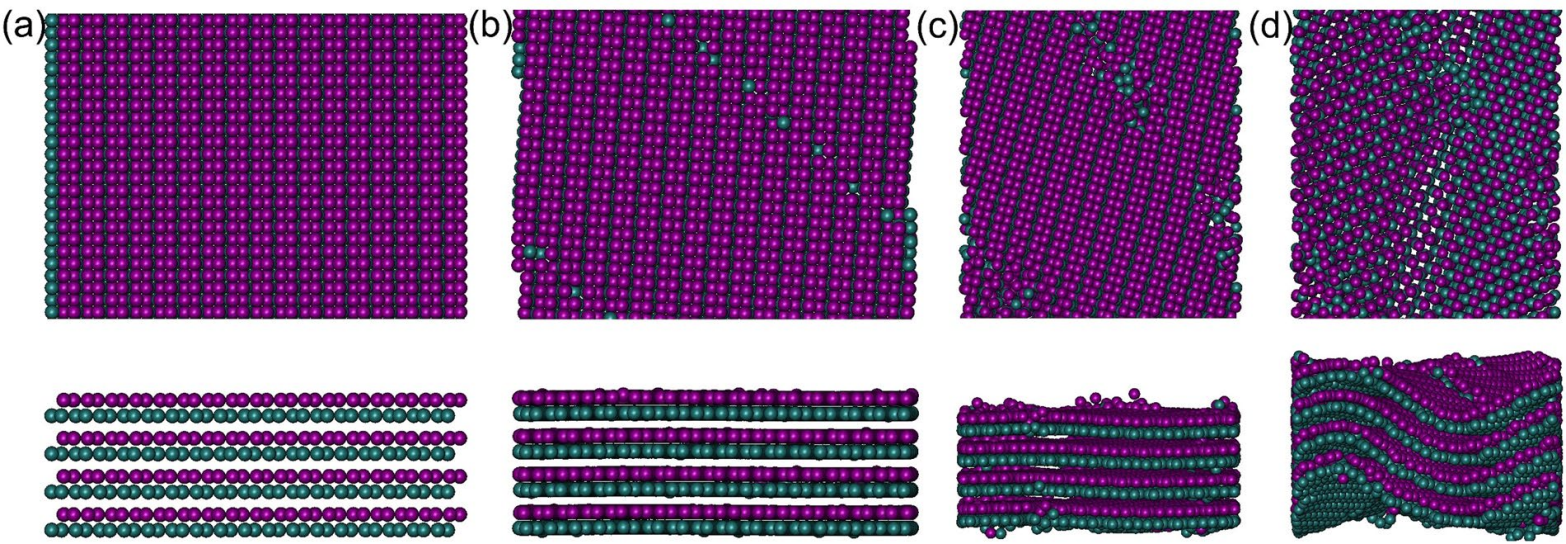
Atomic configurations of four-layer BP (*L*_a_/*L*_b_ = 1.9) at selected compressive strains along the armchair direction: (**a**) ε^−^ = 0.165; (**b**) ε^−^ = 0.230; (**c**) ε^−^ = 0.325; and (**d**) ε^−^ = 0.360. Those in the upper rows are in-plane configurations and ones in below are side views.

Another noticeable observation in Fig. 6a is the occurrence of oscillations with gradually decaying peaks in the stress-strain curves for FLBPs with *L*_a_/*L*_b_ ≥ 1.9 after the strain goes beyond ε^−^ = ~0.230. This can be interpreted from the atomic structure perspective by looking at the atomic configurations in Fig. 7 for the four-layer BP with compressed in the armchair direction. As shown in Fig. 7a, the puckers become very close at compressive strain ε^−^ = 0.165 which corresponds to the point with slope change in Fig. 6a. As the compression further increases to ε^−^ = ~0.230, although the FLBP still remain flats, its armchair lattice loses part of its resistance to such a large compressive loading subsequently starts to deviate slightly from the loading direction as shown in Fig. 7b. In other words, reorientation of FLBP’s atomic structure takes places before buckling happens. At the same time, some vacancies also start to appear in the FLBP. As the compressive strain continues to increase to ε^−^ = 0.325, the stress goes up to a new critical value then suddenly drops again, showing that a further reorientation occurs with more atom vacancies (Fig. 7c). As observed in Fig. 6, this process is repeated three times until the FLBP crushes after the compressive strain further increases to ε^−^ = ~ 0.360. The deformed atomic configuration in Fig. 7d clearly shows that the FLBP is both deflected and twisted at final failure.

The mechanical behavior of the FLBP subjected to a compression along the zigzag direction is quite different from the observations in Figs 6a and 7. This is mainly because its anisotropic puckers in which P atoms are arranged in a zigzag chain-like lattice are capable of sustaining stress higher than the inter-layer interactions so that only bending deformation occurs when the compressive strain reaches a critical value.

**Figure 8:**
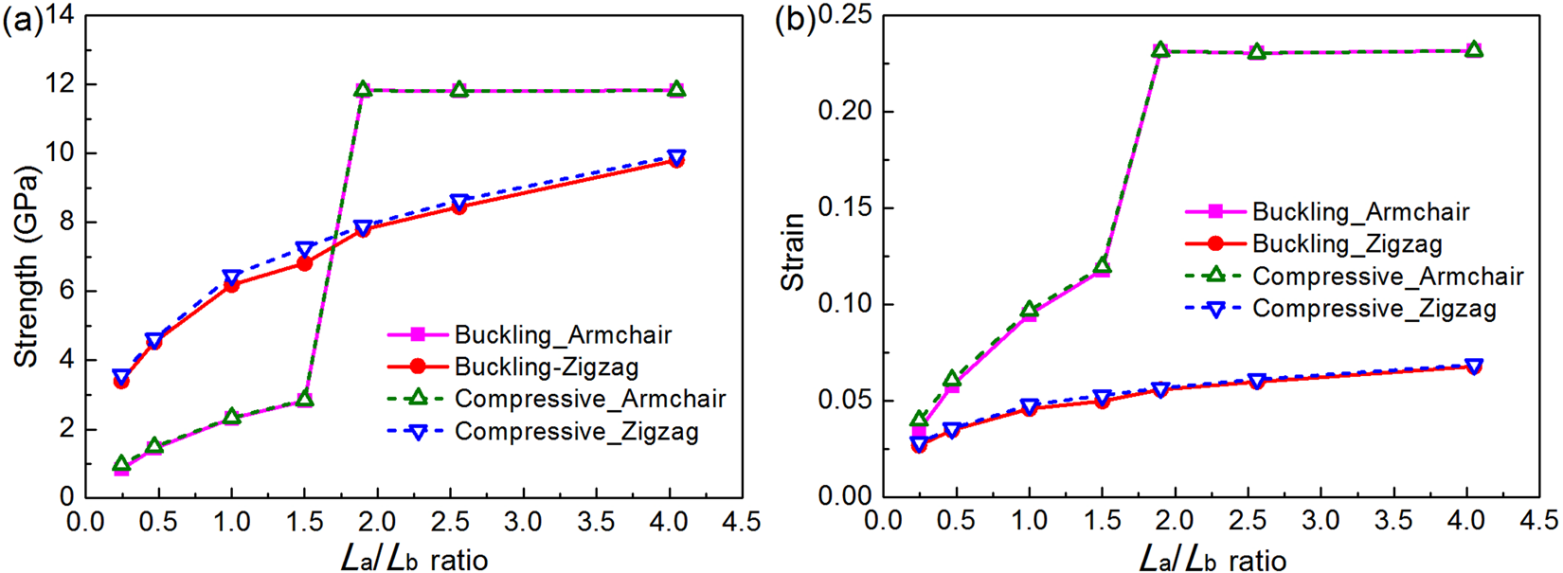
Effect of *L*_a_/*L*_b_ ratio on FLBP’s buckling and compressive strengths/strains in both armchair and zigzag directions: (**a**) compressive strength; and (**b**) compressive strain.

The buckling and compressive strengths and the associated strains in both armchair and zigzag directions of FLBP at different dimension ratios are given in Fig. 8a,b, respectively. It should be mentioned that FLBP’s strength is deteriorated by the reorientation during compression in the armchair direction. Here both the buckling and compressive strengths/strains in the armchair direction are thus defined as the critical stresses/strains at which reorientation of the atomic configuration happens. As can be seen from Fig. 8a, buckling and compressive strengths are improved with increasing *L*_a_/*L*_b_ ratio, indicating that an FLBP with a bigger *L*_a_/*L*_b_ ratio has a better resistance to buckling and compressive failure in both directions. In addition, these strengths in the armchair direction are lower than those in the zigzag direction with the same *L*_a_/*L*_b_ (*L*_a_/*L*_b_ ≤ 1.5) due to the fact that the puckers make the FLBP less stiff in the armchair direction. However, they become higher than their counterparts in the zigzag direction when dimension ratio *L*_a_/*L*_b_ ≥ 1.9, which is consistent with the results in Fig. 6a. Figure 8b shows the similar effect of the *L*_a_/*L*_b_ ratio on both buckling and compressive strains which increase as *L*_a_/*L*_b_ ratio increases. Due to the higher flexibility caused by puckers, the buckling and compressive strains in the armchair direction are always higher than those in the zigzag direction. In summary, increasing the *L*_a_/*L*_b_ ratio is an effective way to achieve significantly improved buckling and compressive strengths/strains of FLBP given that the total number of atoms in the atomic structure is constant. This finding can serve as a guide to predict the maximum stresses the FLBP with different dimension ratios can sustain when designing FLBP based nanodevices.

**Figure 9:**
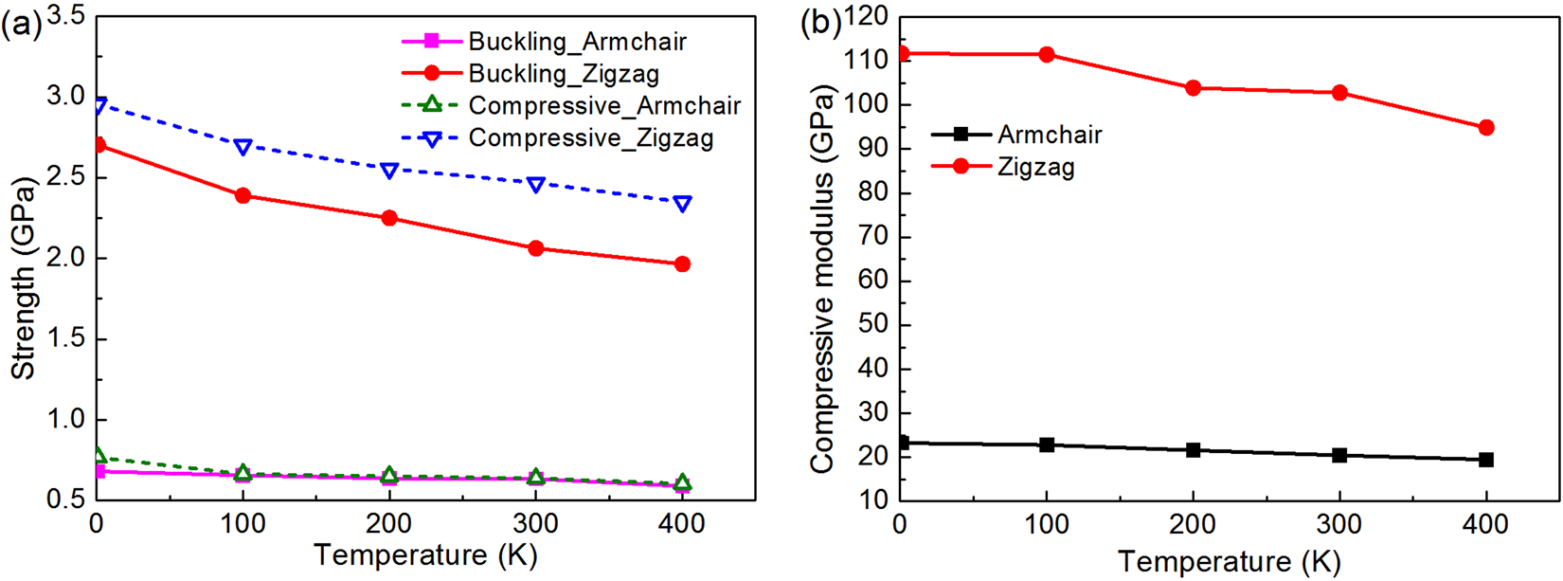
The effect of temperature on (**a**) buckling strength and compressive strength; and (**b**) compressive moduli of BP with two atomic layers.

Figure 9 investigates the effect of temperature on the buckling strength, compressive strength and compressive moduli of an FLBP where the results for a double-layer BP with dimensions (103 Å × 103 Å) are provided. As expected, an increase in temperature leads to reduced strengths and moduli because the atoms in the system gain more energy to overcome the energy barrier at a higher temperature, making the material weaker. This phenomenon has been observed in the previous study on SLBP^24^. Note that the reduction in the properties is slightly different in the two directions and the compressive properties in the zigzag direction is more sensitive to temperature than those in the armchair direction.

### Tensile behaviour

**Figure 10:**
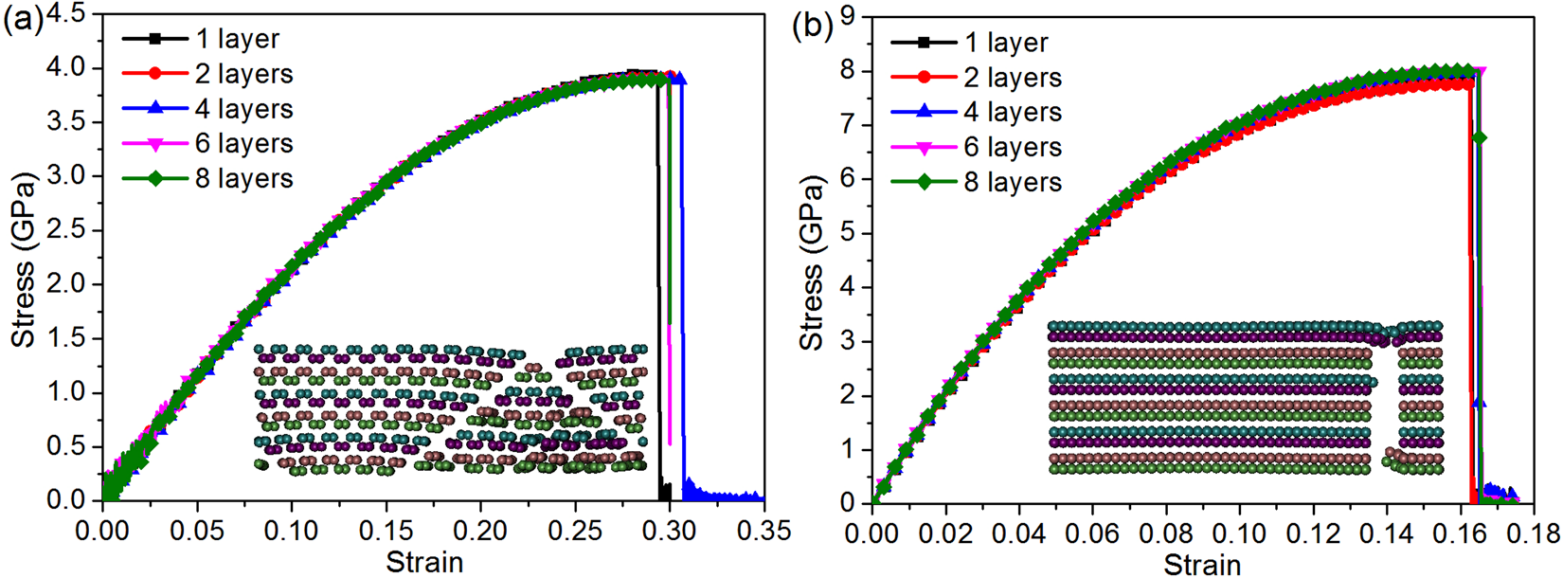
Effect of total number of atomic layers on FLBP’s tensile behavior in the (**a**) armchair direction, and (**b**) zigzag direction. Insets are atomic configurations of fractured six-layer BP.

The tensile behavior of FLBP with different number of atomic layers is examined in this section. Figure 10 gives the tensile stress-strain curves which exhibit quite similar trend in both armchair and zigzag directions, i.e., the tensile stress increases monotonically with an increase in strain then suddenly drops to zero after reaching the maximum values of stress and strain defined as tensile strength and ultimate tensile strain, respectively. This behavior can be ascribed to the typical brittle failure mode^25^, which is clearly demonstrated by the insets in Fig. 10a and b. The tensile strength is found to be ~4.09 GPa with an ultimate tensile strain of ~0.291 in the armchair direction (Fig. 10a) and ~8.42 GPa with an ultimate strain of ~0.162 in the zigzag direction (Fig. 10b). The effect of the total number of atomic layers is insignificant and almost negligible, which is quite different from the compressive behavior that is strongly dependent on how many atomic layers are stacked together in an FLBP.

The tensile modulus can be determined from the slope of the linear region with strain ≤0.01 in the tensile stress-strain curve by using linear regression and results are listed in Table 2. Similar to the compressive modulus in Table 1, the tensile modulus is also insensitive to the total number of atomic layers and is found to be in a range of 23.49~24.34 GPa in the armchair direction and 108.37~108.71 GPa in the zigzag direction, respectively. These simulation results agree well with those in previous *ab initio*^7^ and MD studies^16–18^. As mentioned above, the puckered lattice in the armchair direction offers superior flexibility, giving rise to a large ultimate tensile strain but low strength and tensile modulus whereas the stiffer zigzag chain-like lattice enhances both the tensile strength and modulus but results in a smaller ultimate strain in the zigzag direction.

**Table 2:** FLBP’s tensile moduli (GPa) with different total number of atomic layers.

	1 layer	2 layers	4 layers	6 layers	8 layers
Armchair	24.29	24.34	24.31	23.88	23.49
Zigzag	108.48	108.41	108.53	108.37	108.71

Finally, a direct comparison between the results in Tables 1 and 2 shows that FLBP’s compressive and tensile moduli in the same direction are roughly the same, indicating that FLBP is unidirectional-homogeneous for tensile and compressive deformation in the same direction.

## Conclusions

In this work, the mechanical behaviors of FLBP in both armchair and zigzag directions are investigated through MD simulations, with a particular focus on the effect of total number of atomic layers. It is found that FLBP’s compressive behavior is significantly affected by the total number of atomic layers and crystallographic orientation. For an FLBP with more atomic layers stacked up, its compressive and buckling strengths and strains are considerably enhanced whereas this effect on the Young’s modulus and tensile strength is almost negligible. All compressive and buckling strengths and compressive modulus decrease with increasing temperatures in both two directions. Moreover, the compressive and buckling strengths in the armchair direction, which are lower than those in the zigzag direction for FLBP with the number of atomic layer *N*_*L*_ ≤ 4 or dimension ratio *L*_a_/*L*_b_ ≤ 1.5, significantly increase and become higher than their counterparts in the zigzag direction for FLBP with *N*_*L*_ ≥ 6 or *L*_a_/*L*_b_ ≥ 1.9. It is interesting to observe that the compressive deformation is accompanied by reorientation when the FLBP with *L*_a_/*L*_b_ ≥ 1.9 is under the compression in the armchair direction. However, tensile behavior of FLBP is independent of the total number of atomic layers.
